# Relevance-Based Template Matching for Tracking Targets in FLIR Imagery

**DOI:** 10.3390/s140814106

**Published:** 2014-08-04

**Authors:** Gianluca Paravati, Stefano Esposito

**Affiliations:** Politecnico di Torino, Dipartimento di Automatica e Informatica, Corso Duca degli Abruzzi 24, 10129 Torino, Italy; E-Mail: stefano.esposito@studenti.polito.it

**Keywords:** target tracking, infrared sensors, forward-looking infrared images, template matching

## Abstract

One of the main challenges in automatic target tracking applications is represented by the need to maintain a low computational footprint, especially when dealing with real-time scenarios and the limited resources of embedded environments. In this context, significant results can be obtained by using forward-looking infrared sensors capable of providing distinctive features for targets of interest. In fact, due to their nature, forward-looking infrared (FLIR) images lend themselves to being used with extremely small footprint techniques based on the extraction of target intensity profiles. This work proposes a method for increasing the computational efficiency of template-based target tracking algorithms. In particular, the speed of the algorithm is improved by using a dynamic threshold that narrows the number of computations, thus reducing both execution time and resources usage. The proposed approach has been tested on several datasets, and it has been compared to several target tracking techniques. Gathered results, both in terms of theoretical analysis and experimental data, showed that the proposed approach is able to achieve the same robustness of reference algorithms by reducing the number of operations needed and the processing time.

## 1. Introduction

Detection and tracking of objects and people represent an important research topic in computer vision. The ever-increasing need for automatic, fast and reliable solutions for extracting information from video flows through image processing techniques are dictated by the large domain of vision-based applications. In fact, nowadays, a growing number of applications are envisioned to analyze the motion of pedestrians or moving objects in several scenarios, such as driver assistance (e.g., warning drivers about obstacles on the road, helping with the piloting of aircraft), surveillance and human activity recognition (e.g., locating pinpointing sources of ignition during firefighting operations, the control of servo-motor cameras in security areas), *etc.* From this point of view, image processing techniques are useful, among others, for tracking both vehicles (e.g., in automatic traffic monitoring tools) and people (e.g., for the detection of potentially dangerous situations).

In general, the use of color cameras working in the visible domain represents the most investigated and widespread solution in the above-mentioned applications. However, visible light sensors suffer from illumination issues that make this solution not capable of correctly accomplishing its tasks in all-day and all-weather conditions [1]. The advances in infrared sensor technology laid the ground for introducing the use of forward-looking infrared (FLIR) cameras to overcome some limitations due to the use of traditional color sensors. In fact, thermal-infrared images present a key advantage with respect to images produced by sensing visible light. Since the intensity values are determined by the temperature and radiated heat of objects in the field of view, lighting conditions and object properties, such as material color or texture features, do not influence the generation of the image. However, often, the visible light and the infrared spectrums have been used together in sensor fusion approaches to exploit the benefits of both domains, at the cost of a generally increased system and computational complexity. Moreover, additional challenges are posed when dealing with real-time applications and limited-resource environments. For example, mobile platforms can be equipped with on-board sensors to perform autonomous navigation and tasks [2]; in this scenario, a target-following task is usually implemented by enabling the corresponding actuators after the target detection and tracking phases. Data coming from on-board sensors can be processed and analyzed both locally and remotely [3]. It is worth noticing that, in the case of remotely-processed video flows, the real-time requirements are not only affected by the computational load of the image processing procedure; indeed, the overall latency might be affected by the performance of the communication network. However, proper network technologies exist that guarantee real-time delivery over packet-switched networks. For example, it has been shown that pipeline forwarding technology can offer deterministic delivery over wireless [4], wired [5] or even all-optical [6] networks.

This paper deals with target tracking in forward-looking infrared image sequences; in this context, the thermal imprint of a target is a distinctive feature with respect to background and clutter. Generally, target tracking applications are based on three consecutive steps: first, the detection of stationary or moving objects of interest; then, the tracking of these objects frame by frame; lastly, the classification of the target motion through the analysis of the objects' tracks to recognize their behavior. In this paper, the focus is on the second phase. In particular, the key contribution of this paper is the design of a novel strategy for improving the computational speed of template-matching-based algorithms, the computational domain of which is reduced by selecting a subset of pixels to be analyzed according to a relevance-based strategy.

The designed technique has been compared both to algorithms based on template-matching steps and several other traditional algorithms in target tracking applications. With the aim of assessing the devised technique on different working conditions, experimental tests have been carried out on FLIR image sequences from various datasets; for this reason, both object (vehicle) and pedestrian tracking scenarios have been considered. Results have been gathered both in terms of computational speed and precision. The results obtained by the proposed algorithm indicate an improvement in computational speed by maintaining precision comparable to that achievable by reference algorithms based on traditional template-matching implementation.

The remainder of this paper is organized as follows. Section 2 provides a review of the main target tracking techniques in FLIR imagery. Section 3 focuses on the aspects pertaining to reference algorithms used as the basis for the current work, whereas the proposed solution is presented in Section 4. A detailed theoretical analysis evaluating the use of resources and an evaluation of the tracking performance are illustrated in Section 5. Finally, conclusions are drawn and future research directions are sketched in Section 6.

## 2. Background

Visual tracking is an important topic in computer vision due to the ever-growing number of applications and systems that benefit from its integration, such as traffic monitoring [7] and video surveillance [8]. In spite of many efforts, some challenges remain to be faced in order to build accurate and reliable tracking systems, such as dealing with occlusions, the alternating appearance of objects, illumination issues, and so on. Different techniques have been proposed to cope with different situations. Basically, target tracking can be realized by using traditional color camera sensors, infrared cameras and exploiting data-fusion techniques. Visual tracking with color cameras has been widely investigated. Among the most recent works in this field, studies of interest encompass detection by classification techniques to deal with adaptability for occlusion, appearance and illumination changes [9]. New schemes to account for appearance variation are considered in [10]. Recently, sparse representation has been widely applied to visual tracking as a solution to illumination changes and occlusions [11–14]. However, these techniques are not able to deal with sequences characterized by poor illumination.

Among the numerous algorithms for target tracking, only a limited amount of them is specifically designed to address the particular issues of target tracking in FLIR imagery. Target tracking in infrared images presents several issues. First of all, they present a low signal-to-noise ratio and are affected by the sensors' ego-motion [15,16]. Several techniques have been devised to cope with such a scenario [17–19]. Traditionally, target tracking has been based on two phases: a target detection (TD) step and a target tracking phase based on spatio-temporal correlation, like the mean-shift algorithms [20–22]. Target detection should be realized using very fast techniques, such as the intensity variation function (IVF) [17]. However, some conditions in FLIR images can invalidate the traditional mean-shift algorithms, for instance the assumptions that they are based on might not be true in the case of sensor ego-motion. Moreover, the presence of similar target signatures or noise represent conditions that lead the TD to fail in FLIR images, returning wrong results; hence the necessity of a strategy for the activation of a recovery phase [23].

To cope with a low signal-to-noise ratio and sensor ego-motion, target correlation approaches have been explored. Among target correlation-based techniques, the one presented in [17] has the peculiarity of using a small and compact target signature for fast frame-to-frame target tracking through IVF, using a larger template only to recover from IVF failures through a template matching (TM) technique. The higher reliability of TM is in terms of resources usage, which is much more intense in the TM phase with respect to the TD phase. In other words, TM is slower than TD, even though it is able to generate more precise results.

The reference algorithms selected for this work, presented in [17,18], are based on the TM technique. Although, while in [17], the IVF failure detection was based on a Cartesian distance metric, in [18], a motion prediction-based metric is presented, and it showed better tracking performances than the Cartesian metric. The techniques presented in [17,18] are analyzed more in detail in Section 3, since they constitute the base layer of the proposed algorithmic improvement.

## 3. Reference Algorithms

The target tracking procedure followed in this work puts down its roots in the techniques proposed in [17,18], in the following, referred to as ATT (automatic target tracking) and PATT (predictive automatic target tracking), respectively. Both of them have been employed as a reference for evaluating the performance of the proposed solution in Section 5.

Both ATT and PATT use a target detection (TD) phase and a possible target recovery phase; the TD phase is based on the IVF algorithm, and the eventual recovery phase is based on a template matching algorithm. TM is triggered when false alarms are detected during the TD phase. In particular, the detection of IVF false alarms is performed through two different strategies. In [17], a Cartesian distance metric approach is used, while in [18], a motion prediction-based metric and a probabilistic evaluation is introduced.

In the following paragraphs, the main concepts concerning the TD and recovery phases are reviewed. These concepts will be recalled in Section 4 during the exposition of the proposed algorithm.

### 3.1. IVF-Based Target Detection

The detection of targets is based on the analysis of their thermal signature. This phase exploits a local maximum window extracted from the previous frame to compute IVF and uses IVF results to find a new local maximum representing the candidate target position for the current frame. Computations are limited to a sub-frame to avoid non-target objects from the background being identified as potential targets by the algorithm. This simplification clearly assumes that the target motion among frames is confined within the sub-frame. IVF is defined as follows.

(1) $$F^{n}(\upsilon ,z)=\frac{1}{\Lambda }\sum_{j=0}^{l}\sum_{i=0}^{k}\left[S^{n}(i+\upsilon ,j+z)-\omega^{n-1}\right]$$

In Equation (1), Λ = *k* × *l* is the area of the target window, *υ* and *z* are the coordinates in the sub-frame, *ω^n^*^−1^ is the local maximum matrix in the previous frame (*n* − 1), *S^n^* is the target window centered at (*i* + *v*, *j* + *z*) and *F^n^*(*υ*, *z*) is the IVF computed in (*υ*, *z*) for the current frame *n*. A correlation output plane (COP) is built starting from IVF, and it is defined as follows.

(2) $$C(\upsilon ,z)=e^{-\lambda F^{n}(\upsilon ,z)}$$

In Equation (2), λ is an arbitrary parameter, selected to ensure a satisfactory enhancement of IVF results. The position of the candidate target is associated with the position of the highest peak on the correlation output plane. In fact, the maximum on the COP is by definition the point in the sub-frame most similar to the local maximum in the previous frame; for this reason, it is considered the best candidate to represent the target in the current frame.

**Figure 1.**
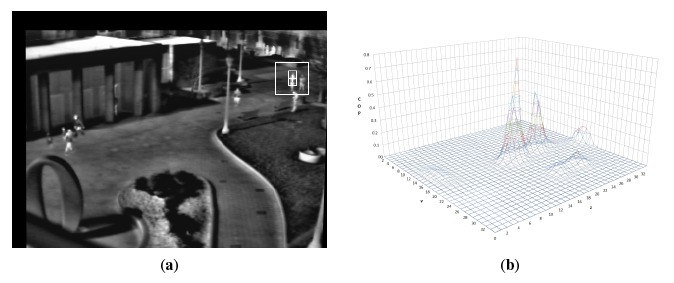
Processing of the intensity variation function (IVF) algorithm in a sample frame from the OTCBVS (Object Tracking and Classification Beyond the Visible Spectrum) dataset [24] (sequence otcbvs 03-l1s2ir-4). (**a**) Frame 57; (**b**) correlation output plane (COP) of Frame 57.

Figure 1 shows an example of the correlation output plane generated by IVF computed on a sample frame extracted from the OTCBVS (Object Tracking and Classification Beyond the Visible Spectrum) dataset [24]. As shown in this example, more peaks with a different local maximum can coexist in the COP. Despite the similarity between local maximum matrices in consecutive frames, it is not guaranteed that the highest peak in the plane (the one selected by IVF) is the real target. In this case, the activation strategy later described determines the need for the execution of a recovery phase. In general, IVF is a fast and reliable algorithm when the sequence is not affected by severe sensor ego-motion and target feature changes are not too swift. Nonetheless, it may be misled by a non-target object included in the sub-frame whose features are similar to those of the target. Moreover when ego-motion is dramatic, the chances of gathering correct results from the IVF algorithm alone are quite low because, due to its small target window and to the low signal-to-noise ratio of the images, a significant sensor ego-motion is very likely to be introduced into the sub-frame of an object with a higher IVF value than the target. Finally, changes in frame features may result in target feature changes, such that IVF is not able to determine the correct position of the target in the current frame. To solve these issues, the detection strategy described in the following section is used to decide when to launch a TM phase able to recover from this type of error.

### 3.2. Cartesian Distance Metric

Despite the good results and proven efficiency of IVF, the low signal-to-noise ratio and occasional sensor ego-motion can lead to wrong matches; a strategy to detect and correct false alarms is therefore mandatory in target tracking for FLIR images. In [17], an approach based on Cartesian distance was used. The algorithm evaluates the distance between the IVF candidate target $p_{\text{IV F}}^{n}$ and the previous position of the target *p^n^*^−1^. Whenever the distance exceeds a threshold *β*, the value of which depends on the sequence features (e.g., the sensor's ego-motion), the TM recovery phase is activated. The distance is computed as follows:

(3) $$d_{\text{IV F}}=\sqrt{{\left(x_{p_{\text{IV F}}^{n}}-x_{p^{n-1}}\right)}^{2}+{\left(y_{p_{\text{IV F}}^{n}}-y_{p^{n-1}}\right)}^{2}}$$

Even though this approach is rather simple and efficient, because little overhead is added for the activation strategy, it might not be effective enough. Indeed, an optimal *β* value is difficult to determine, because it hugely depends on sequence features, such as the motion of the target or the ego-motion of the sensor itself.

### 3.3. Motion Prediction-Based Metric

The strategy proposed in [18] for the activation of the recovery step is based on the target history. Information on the target position in previous frames is stored to generate a motion vector and to elaborate a prediction for the current frame using a position estimator. The candidate target position ${\bar{p}}_{\text{IV F}}^{n}$, computed by IVF, is associated with a motion vector (*p^n^*^−1^, ${\bar{p}}_{\text{IV F}}^{n}$), and it is compared to the predicted motion vector (*p^n^*^−1^, *p̂^n^*) where *p̂^n^* is the target position in the current frame estimated by a linear predictor. The reliability of the IVF result is then evaluated using a conditioned probability approach based on the distance and angle of the motion vectors. In particular, the probability that the result of the target detection phase is the correct target in the current frame is computed as follows:

(4) $$P({\bar{p}}_{\text{IV F}}^{n})=P(d_{\text{IV F}}\cap \alpha_{\text{IV F}})=P(d_{\text{IV F}})\times P(\alpha_{\text{IV F}}|d_{\text{IV F}})$$

where *d_IV F_* is the IVF motion vector length and *α_IV F_* is the angle it describes with the predicted motion vector. In Equation (4), *P* (*d_IV F_*) and *P* (*α_IV F_*∣*d_IV F_*) are defined as follows:

(5) $$P(d_{\text{IV F}})=\{\begin{array}{ll} 1-\frac{\hat{d}-d_{\text{IV F}}}{\hat{d}} & \text{if}\;d_{\text{IV F}}<\hat{d},\\ 1-\frac{d_{\text{IV F}}-\hat{d}}{d_{\text{max}}-\hat{d}} & \text{if}\;\hat{d}<d_{\text{IV F}}<d_{\text{max}}\\ 1 & \text{if}\;d_{\text{IV F}}=\hat{d}\\ 0 & \text{if}\;d_{\text{IV F}}=d_{\text{max}} \end{array}$$

(6) $$P(\alpha_{\text{IV F}}|d_{\text{IV F}})=\{\begin{array}{ll} \frac{\left|\alpha_{\text{IV F}}-180{}^{\circ}\right|}{180{}^{\circ}}\times \left(1-\frac{d_{\text{max}}-d_{\text{IV F}}}{d_{\text{max}}}\right) & \text{if}\;\alpha_{\text{IV F}}\neq 0,d_{\text{IV F}}>0\\ 1 & \text{otherwise} \end{array}$$

Both in Equations (5) and (6), *d_max_* is the maximum distance at which the target can be found given a certain sub-frame size. In Equation (5), *d̂* is the predicted motion vector length; moreover, the probability is defined, so that it is highest if the length of the IVF motion vector is the same as the one of the predicted motion vector; on the other hand, it decreases as the difference between the motion vectors increases, until it is set to zero when distance *d_max_* is reached. In Equation (6), the angular contribution is computed modulo 180°, and it is weighted to minimize its impact on short motion vectors. This probability value is then compared to a confidence level *μ* to decide whether the IVF position should be selected as the new target position (when $P\left({\bar{p}}_{\text{IV F}}^{n}\right)>\mu$) or if a recovery algorithm should be invoked to recover from an error condition (when $P\left({\bar{p}}_{\text{IV F}}^{n}\right)\leq \mu$).

### 3.4. Template Matching

Whenever an error condition is detected through the aforementioned metrics (Sections 3.2 and 3.3), a TM phase is necessary to recover from the error in the TD phase and to find the correct target position on the current frame. The algorithm used in [17,18] is very similar to the described IVF, and it is defined as follows.

(7) $$T^{n}(\upsilon ,z)=\frac{1}{\Phi }\sum_{j=0}^{p}\sum_{i=0}^{m}\left[S^{n}(i+\upsilon ,j+z)-W^{n-1}\right]$$

In Equation (7), *T^n^*(*υ*, *z*) is the computed TM value for the point of coordinates (*υ*, *z*), Φ = (*p* × *m*) is the area of the target window, while *W^n^*^−1^ is the target window in the previous frame. All other symbols have the same meaning as in Equation (1). With reference to Equations (1) and (7), usually *p* > *l* and *m* > *k*, so Φ > Λ; hence the greater resource demand of TM over IVF. As in IVF, the value resulting for each point of the sub-frame is used to build a COP as follows.

(8) $$C_{TM}(\upsilon ,z)=e^{-\lambda T^{n}(\upsilon ,z)}$$

As in the case of IVF, λ is an arbitrary parameter. The highest peak on the COP is taken as the best candidate for the target position on the current frame. Even though the use of *W* instead of the smaller *ω* matrix increases the computational complexity of TM with respect to IVF, it also guarantees the use of information on target shape and surrounding background, allowing one to better discriminate between target and non-target objects. In this way, TM can recognize the target, even when IVF fails. Indeed, TM has a better capability of finding the target at the cost of a considerably higher computational time. This is due to the bigger size of the matrices used in TM computation with respect to the size of the matrices used in IVF computation. In [18], the position ${\bar{p}}_{TM}^{n}$ obtained by the TM phase is then used to build a new motion vector (*p^n−^*^1^, ${\bar{p}}_{TM}^{n}$), which is, in turn, compared to the predicted motion vector (*p^n−^*^1^, *p̂^n^*), as described in Section 3.3. The probability $P\left({\bar{p}}_{TM}^{n}\right)$ is then compared to the probability $P\left({\bar{p}}_{\text{IV F}}^{n}\right)$ previously computed. The point with the highest probability in this comparison is finally selected as the position of the target in the current frame. This step is necessary to avoid drifting issues that can be introduced by TM [18].

## 4. Proposed Algorithm

The computational complexity of the template matching step could undermine the applicability in real-time systems of algorithms using the approach so far described. Indeed, the TM step, as described in Section 3.4, necessarily considers a larger domain of computations leading to long execution times. Therefore in this section, an approach based on the relevance of the sampling points in the sub-frame is proposed with the aim of reducing the computational complexity of the TM step.

The proposed solution for tracking targets in FLIR imagery combines the algorithms presented in Section 3.4 and improves the computational speed of the template matching step. The overall tracking algorithm is presented in Algorithm 1. For each new frame of a sequence, the IVF algorithm computes a candidate target; based on the history of the locations of the target under analysis, an expected target position based on a predictive step is computed, and a probability score is thus associated with the candidate target of the IVF step. Since the IVF algorithm has a small footprint, if the result coming from this step satisfies a minimum confidence level, the location of the candidate target is considered reliable, and it is designated as the current position of the target. Otherwise, additional steps are required to solve the ambiguity. In this case, the template matching procedure involving the analysis of the sub-frame should be activated to gather more accurate results. The correlation output plane is thus analyzed to select an adaptive and suitable threshold used to restrict the computational domain of the TM step. The selected subset of the correlation output plane identifies the areas where the correlation between the searched target and the pixel areas of the current frame is strongest. Within only the selected subset, a score is computed for each possible candidate point, as well as the associated TM value and probability value; as will be explained in more detail later in this section, the score is based both on the TM value and on the likelihood associated with the prediction step for the candidate point under analysis. The higher the threshold, the greater the computational savings. However, it is imperative to avoid too restrictive results; this is the reason why the threshold is designed to be also dynamic: it starts from a high value and decreases as needed to accommodate adequate results (*i.e.*, the scores should satisfy minimum requirements in terms of quality). The three results within the selected subset maximizing the designed score, the TM value and the probability value are finally evaluated with a weighted comparison to choose the new position of the target. Deeper details are given in the remainder of this section. Given the considerable number of symbols cited in the text, for a quick reference, the interested reader can find a digest of them in Table 1.

The traditional TM phase (described in Section 3.4) computes the TM values for each point of the sub-frame, regardless of the likelihood of that point belonging to the target area. The proposed algorithmic improvement takes into account the relevance, *i.e.*, the likelihood of belonging to the target, of a point with coordinates (*υ*, *z*) in the sub-frame before computing *T^n^*(*υ*, *z*) with Equation (7). The relevance of a point is evaluated by comparing the value associated with the point on the COP computed by IVF in the TD phase, using Equation (2), with a threshold *δ*. The threshold is designed to be adaptive on a frame-by-frame basis, and it is dependent on the maximum value on the same COP. A point within the sub-frame is labeled as relevant (*ṗ^n^*) if its value on the COP is above the *δ* threshold. Therefore, the TM function described in Equation (7) is computed only for relevant points of the COP; a detailed discussion about the savings in computational complexity is provided in Section 5.3.



**Algorithm 1** The proposed algorithm (see Table 1 for symbols meaning). Details on weighted comparisons are given in Equations (10)–(13).
**Input:**
*ω^n^*^−1^, *p^n^*^−1^, *history***Output:**
*p^n^*1:*COP* ← *ComputeCOP* (*p^n^*^−1^, *ω^n^*^−1^)2:
${\bar{p}}_{\text{IV F}}^{n}\leftarrow \text{position of}\;\text{max}\{\text{COP}\}$3:*p̂^n^* ← *Linear Prediction*(*history*)4:
$P_{\text{IV F}}\leftarrow P\left({\bar{p}}_{\text{IV F}}^{n}\right)$5:**if**
*P_IV F_* > confidence level **then**6: 
$p^{n}\leftarrow {\bar{p}}_{\text{IV F}}^{n}$7:**else**8: *δ* ← 0.959: **repeat**10:  **for all**
*p* : *C^n^*(*p*) ≥ *δ* × *max*{*COP*} **do**11:   **if**
*maxscore* < *ψ*(*p*) **then**12:    *maxscore* ← *ψ*(*p*)13:   **end if**14:   **if**
*maxTM* < *T^n^*(*p*) **then**15:    *maxTM* ← *T^n^*(*p*)16:    
${ṗ}_{TM}^{n}\leftarrow p$17:   **end if**18:   **if**
*maxP* < *P* (*p*) ∨ *maxPScore* < *ψ*(*p*) **then**19:    *maxP* ← *P* (*p*)20:    
${ṗ}_{P}^{n}\leftarrow p$21:   **end if**22:  **end for**23:  **if**
*WeightedComparison*_*P*_*TM*
$\left({ṗ}_{P}^{n},{ṗ}_{TM}^{n}\right)$
**then**24:   
$p^{n}\leftarrow {ṗ}_{P}^{n}$25:  **else**26:   **if**
*W eightedComparison*_*IV F* _*T M*
$\left({\bar{p}}_{\text{IV F}}^{n},{ṗ}_{TM}^{n}\right)$
**then**27:    
$p^{n}\leftarrow {\bar{p}}_{\text{IV F}}^{n}$28:   **else**29:    
$p^{n}\leftarrow {ṗ}_{TM}^{n}$30:   **end if**31:  **end if**32:  *δ* ← *δ* − 0.233: **until**
*maxscore* ≥ *∊* ∨ *δ* ≤ 034:**end if**


For each relevant point *ṗ^n^*, a motion vector (*p^n^*^−1^, *ṗ^n^*) is computed in order to be compared with the predicted motion vector (*p^n^*^−1^, *p̂^n^*) following Equation (4). The likelihood value *P*(*ṗ^n^*) resulting from the comparison of motion vectors is used along the value resulting from the computation of the template matching value *T^n^*(*ṗ^n^*) for the same point with the aim of defining a score as follows:

(9) $$\psi ({ṗ}^{n})=P({ṗ}^{n})\times T^{n}({ṗ}^{n})$$

where *ψ* (*ṗ^n^*) is the score associated with a relevant point *ṗ^n^*. To ensure that a significant number of relevant points is found for a specific frame, *i.e.*, the subset is not too small, a minimum score *∊* is required. If no point in the current subset reaches the required minimum score *∊*, the threshold *δ* is lowered, so that other relevant points can be added to the subset. Once the set of points is considered large enough, *i.e.*, when at least one of the points in the subset reaches the required minimum score ∊, the algorithm should decide which of these points represents the target in the current frame. The point with the highest probability ${ṗ}_{P}^{n}$ and the point with the highest TM value ${ṗ}_{TM}^{n}$ are subjected to a weighted comparison to decide whether to choose ${ṗ}_{P}^{n}$ or not. The Boolean function performing the decision is represented in the following:

(10) $$a\times (bc+\bar{b}d+e)$$

In Equation (10), a weight *α* is used for the TM value, and a weight *β* is used for the probability one. In particular,

(11) $$\begin{array}{l} a=T^{n}\left({ṗ}_{P}^{n}\right)+\alpha >T^{n}\left({ṗ}_{TM}^{n}\right)\\ b=P\left({ṗ}_{P}^{n}\right)<0.9\\ c=P\left({ṗ}_{P}^{n}\right)-\beta >P\left({ṗ}_{TM}^{n}\right)\\ d=P\left({ṗ}_{P}^{n}\right)>P\left({ṗ}_{TM}^{n}\right)\\ e=P\left({ṗ}_{TM}^{n}\right)+\eta <P\left({\bar{p}}_{\text{IV F}}^{n}\right) \end{array}$$

When the Boolean function (10) returns a true value, ${ṗ}_{P}^{n}$ is selected as the position for the target in the current frame; otherwise ${ṗ}_{TM}^{n}$ is compared to the IVF candidate position ${\bar{p}}_{\text{IV F}}^{n}$. Experimental results in Section 5 have been gathered using *η* equal to *α*; moreover, the required level of confidence in the probability metric was set to 0.9.

Likewise, the following Boolean function has been designed to choose between ${ṗ}_{TM}^{n}$ and ${\bar{p}}_{\text{IV F}}^{n}$:

(12) f×(g+h)×i

where:

(13) $$\begin{array}{l} f=\psi ({\bar{p}}_{\text{IV F}}^{n})>0\\ g=\psi ({\bar{p}}_{\text{IV F}}^{n})+\iota >\psi ({ṗ}_{TM}^{n})\\ h=T^{n}({ṗ}_{TM}^{n})-T^{n}({\bar{p}}_{\text{IV F}})<0.15\\ i=P({\bar{p}}_{\text{IV F}}^{n})+\xi >P({ṗ}_{TM}^{n}) \end{array}$$

In Equation (12), *ξ* is numerically equivalent to *ι* in the performed tests. In this case, when the Boolean function (12) returns a true value, ${\bar{p}}_{\text{IV F}}^{n}$ is selected as the position of the target in the current ${ṗ}_{TM}^{n}$ frame; otherwise is selected. Weights in the above equations are assigned so that the correlation is a main criterion for the selection of the target position; on the other hand, also the motion prediction-based metric is taken into account to make sure that the best possible choice is made.

Moreover, the candidate points obtained from the IVF step are always reconsidered against the TM preferred points as in [18]; this is to avoid the TM being subject to drifting and losing the target, as happens when only TM routines are used, without an IVF-based target detection phase. Overall, the logic represented in Equations (10) and (12) showed a satisfactory robustness at the cost of using some thresholds. These levels of confidence depend on sequence features and on the desired precision of the algorithm.

## 5. Results and Discussion

The performance of the proposed algorithm in terms of tracking speed has been evaluated, both from a theoretical and experimental point of view. In fact, the primary objective of the relevance-based algorithm is to enhance the computational efficiency by discarding useless computations in areas with a low probability of finding a target. A preliminary theoretical analysis of the improvements introduced by adopting the devised technique has been carried out with respect to the reference algorithms ATT [17] and PATT [18], on which this work is based. For this purpose, a set of metrics has been designed in Section 5.1 to enable a fair comparison between these algorithms. Moreover, the analysis of the tracking speed has been widened by taking into account several alternative and faster algorithms according to a recent benchmark on online tracking [25].

### 5.1. Assessment Criteria

With the aim of evaluating the computational efficiency of the devised technique, this section introduces the metrics designed to make a comparison with the reference tracking methods [17,18].

As previously described, all the considered algorithms share the computation of the template matching Function (7), which is activated when tracking error conditions are met. In reference algorithms, the template matching function *T^n^*(*P_e_*) is computed for each evaluation point *P_e_*(*υ, z*) with coordinates (*υ*, *z*) lying inside the sub-frame. For each evaluation point Φ subtractions, Φ − 1 additions, one division and one exponentiation are required, where Φ is the target window area, as defined in Section 3.4. The complexity of the implementation is therefore *O*(Φ) for reference algorithms.

The devised technique proposes to execute the *T^n^*(*P_e_*) function on a subset of points of the sub-frame. The operations executed on each relevant point are: Φ subtractions, Φ − 1 additions, one division, one multiplication and one computation of probability. Since the algorithm implementing the probability computation has a complexity *O*(1), the overall complexity of the proposed implementation is also *O*(Φ); from these considerations, it follows that the size *S*_{*P_e_*}_ of the domain of evaluated points *P_e_* can be assumed as a valid comparison metric to evaluate the complexity savings of the proposed algorithm with respect to the reference algorithms.

In the reference algorithms, the size of the domain of evaluated points directly depends on the number of template matching activations *m*, and it is proportional to the size of the target window during the target detection phase:

(14) $$S_{\left\{P_{e}\right\}}=\Lambda \times m$$

On the other hand, the size of the domain of evaluated points with the relevance-based algorithm is defined as follows:

(15) $$S_{\left\{P_{e}\right\}}=|\{p:C^{n}(p)\geq \delta \}|$$

The comparison of the domain size of the template matching function is useful for giving an idea of the boost in performance, as discussed in Section 5.3. However, since it relates to only a portion of the overall tracking algorithm, it is necessary to introduce also another metric able to take into account the most relevant parts of the target tracking algorithms. With this intent, it is worth defining the number of operations required by the different algorithms, including IVF execution from the first to the last frame of a sequence. Since sum and subtraction operations are dominant in both the reference and proposed implementations, the designed metric is based on the number of these operations. IVF requires a number of operations dependent on the size of its target window Λ; in particular, it requires Λ subtractions and Λ − 1 additions. Similarly, the TM phase requires Φ subtractions and Φ − 1 additions; thus, the number of operations of this kind performed by reference algorithms is computed as follows:

(16) Θ(n,m)=n×[Ψ×(2Λ−1)]+m×[Ψ×(2Φ−1)]

where *n* is the number of frames in the sequence, *m* identifies the number of activations of the TM phase in the sequence, Ψ is the sub-frame area and Λ is the IVF target window area. The number of operations for the reference algorithms can be computed with the same Equation (16), because it is not dependent on the activation strategy, and the same TD and TM phases are used by both algorithms.

Similarly, the number of operations performed by the proposed algorithm is computed as follows:

(17) Ω(n,p)=n×[Ψ×(2Λ−1)]+p×(2Φ−1)

In Equation (17), *p* is the number of relevant points found and analyzed throughout the sequence, whereas the other symbols have the same meaning as Equation (16). It is worth noticing that, in both formulas, a double contribution is considered: the first product takes into account IVF operations, whereas the second product is related to the TM phase.

### 5.2. Analysis of Computational Complexity

The proposed algorithm, in the following referred to as RATT (relevance-based ATT), and the reference ones have been tested on a set of FLIR sequences to measure the designed metrics and to perform an analysis of their computational complexity. Sequences from various public datasets have been considered to take into account different target shapes, background scenarios and sensor and image characteristics (such as resolution). The considered datasets include the OTCBVS 03/OSU (Ohio State University) Color and Thermal Database [24], the Army Missile Command (AMCOM) FLIR dataset and the AIC (Adaptive Information Cluster) Thermal/Visible Nighttime Database [1,26]. The first and the latter concern the tracking of pedestrians, whereas the second one represents a database of military sequences.

**Figure 2.**
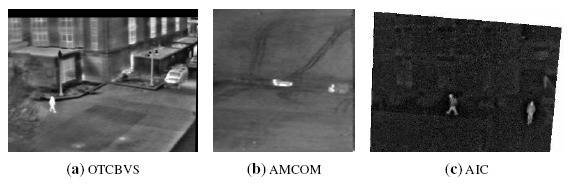
Excerpts from the considered dataset. (**a**) OTCBVS (Object Tracking and Classification Beyond the Visible Spectrum) sequence; (**b**) Army Missile Command (AMCOM) sequence; (**c**) AIC (Adaptive Information Cluster) Thermal Database sequence.

Figure 2 shows some excerpts taken from the aforementioned datasets. Sequences from the OTCBVS 03/OSU Color and Thermal Database have a resolution equal to 320 × 240 pixels. the AMCOM sequences are provided at a resolution equal to 120 × 120 pixels, and the AIC Thermal/Visible Nighttime frames have a resolution of 640 × 480 pixels. In all of the considered datasets, test sequences have been extracted in such a way that target losses do not occur by using reference algorithms. In particular, twelve sequences have been extracted, both from the OTCBVS and AMCOM datasets, and two sequences have been considered for the AIC database. For each sequence, one or more particular subset of frames has been identified to isolate the appearance and disappearance of targets; in fact, the coordinates and size of the target have been provided to the algorithms for the first frame of each sequence.

Both the reference and the proposed algorithms have been executed on the datasets using the same common parameters. In particular, all of the three algorithms share the same sub-frame size, target window size and the initial target position. The target window size is different for each sequence, since it depends on the shape of the target itself; on the other hand, the sub-frame has been kept constant with a size equal to 33 × 33 pixels, as indicated in [17] for the first two datasets (OTCBVS and AMCOM). A slightly wider sub-frame (44 × 44 pixels) has been used with the AIC dataset for enabling a complete encapsulation of the target window inside the sub-frame. Instead, concerning only the comparison with the PATT algorithm, the same level of confidence *μ* has been used to trigger the activation of the TM phase.

Table 2 reports the results for all of the above-mentioned sequences concerning the number of activations of the TM phase and the number of evaluated points. The first three columns provide the identifier of the sequence (Seq.), its length *L* (expressed in frames) and the size of the target window *S_TW_*, respectively. The fourth and fifth column show the number of activations *m* of the TM phase and the size of the domain of evaluated points *S*_{*P_e_*}_ for the ATT algorithm. Similarly, the next four columns provide the same information for the PATT algorithm and the proposed one, respectively. Finally, the last two columns give an indication of the behavior of the proposed algorithm with respect to ATT (second to last column) and PATT (last column), in terms of the variation of the size of the domain of evaluated points. More specifically, they indicate the percentage of points for which the template matching function *T^n^*(*P_e_*) is evaluated by using Equation (7) for the proposed algorithm with respect to the ones evaluated by the reference techniques. *S*_{*P_e_*}_ is computed by using Equations (14) and (15).

The theoretical analysis shows that, in general, it is possible to hugely reduce the size of the function domain despite a higher number of TM activations that are triggered by the algorithm. In fact, in most cases, the number of evaluated points is a small percentage of the size of the original domains. For example, let us consider sequence otcbvs 03-l2s6ir-3: a very high number of activations occur using the proposed algorithm (template matching is triggered 200-times, about two thirds of the length of the sequence); on the other hand, ATT requires only eight activations, and PATT requires 124 activations. Nevertheless, the proposed technique really evaluates the template matching function on 15.86% of the points with respect to ATT and only on 1.02% of the points with respect to PATT.

The different number of observed activations among algorithms is due to the different results given by the respective template matching processes. In this way, the history of target positions slightly changes and different probabilities are computed, which, in turn, are used to determine the activation of the TM phase. In some cases, reference algorithms never trigger any recovery phase.

**Figure 3.**
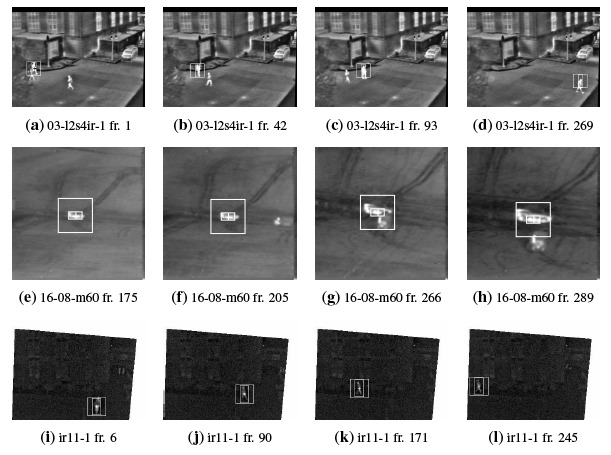
Tracking results by running the proposed algorithm on sample sequences extracted from each considered dataset. (**a**–**d**) OTCBVS sequence 03-l2s4ir-1; (**e**–**h**) AMCOM sequence 16-08-m60; (**i**–**l**) AIC ir11-1.

Figure 3 visually shows the tracking results by running the proposed algorithm on a sequence for each dataset. Since the behavior of ATT and PATT from the point of view of the tracked position is analogous to RATT, their frames are omitted. The OTCBVS dataset is represented by the frames extracted from the sequence 03-l2s4ir-1 in Figure 3a–d, where pedestrians are tracked throughout the sequence. Vehicles are considered in the AMCOM dataset; an excerpt of these tests is provided by sequence 16-08-m60 in Figure 3e–h. Finally, Figure 3i–l concerns again pedestrian tracking (sequence ir11-1). The smallest rectangle represents the bounding box (*i.e.*, the target window) of the target of interest; the widest one represents the search area (*i.e.*, the sub-frame).

**Figure 4.**
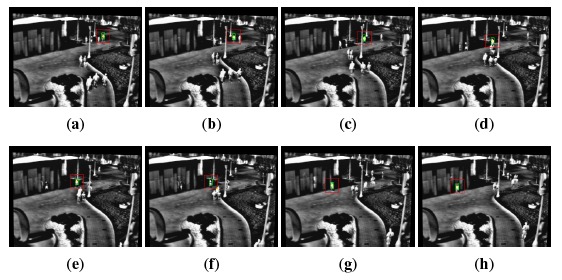
Tracking results with the ATT algorithm for sequence otcbvs 03-l1s1ir-12. (**a**) Frame 142; (**b**) Frame 188; (**c**) Frame 249; (**d**) Frame 333; (**e**) Frame 391; (**f**) Frame 407; (**g**) Frame 498; (**h**) Frame 556.

**Figure 5.**
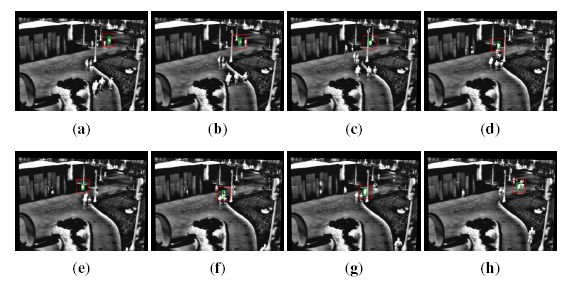
Tracking results with the proposed algorithm for sequence otcbvs 03-l1s1ir-12. (**a**) Frame 142; (**b**) Frame 188; (**c**) Frame 249; (**d**) Frame 333; (**e**) Frame 391; (**f**) Frame 407; (**g**) Frame 458; (**h**) Frame 556.

It is worth pointing out that, in some cases, the proposed computational savings can come at the expense of the tracking robustness. As anticipated before, sequences have been selected in such a way that target losses do not occur with the reference algorithms; in Table 2, *F* indicates a failure in the tracking algorithm. Considering the extended dataset, the proposed algorithm gets into a target loss for the sequence otcbvs 03-l1s1ir-12; more in detail, in this case, only 64% of the sequence has been correctly tracked. For this reason, the comparison of the number of evaluated points is not meaningful; therefore, in Table 2, it is not reported. On the other hand, all of the other sequences are tracked successfully. Figures 4 and 5 point out significative frames for the sequence otcbvs 03-l1s1ir-12 using the ATT and the proposed algorithm, respectively. This sequence represents a challenging situation, due to the presence of similar targets in the scene. Indeed, though the behavior of the original algorithm is correct (Figure 4), a tracking failure occurs with the proposed technique (Figure 5); in this case, from (a) to (e), the tracking is correct; from (f) to (h), the algorithm selects an improper peak in the correlation output plane, thus giving rise to the failure.

The first designed metric gives only an indication of the possibility of reducing the computations. With the aim of better evaluating the complexity of the considered algorithms, Tables 3 and 4 summarize the results of the theoretical analysis, introducing the comparison of the number of estimated operations among the same algorithms considered so far. Moreover, the theoretical analysis is complemented with real average speed at run-time. The dominant operations in all considered algorithms are the sums and subtractions performed by IVF and TM; thus, Table 3 shows the number of such operations performed by all considered algorithms as described in Section 5.3. Due to the different kinds of parameters involved, the number of operations Θ with ATT and PATT are estimated by using Equation (16) and the number of operations Ω of the reference algorithm are computed with Equation (17). After listing the number of operations and the average time per frame *T_A_* for each algorithm, the four columns of Table 4 provide a comparison of the proposed algorithm with ATT and with PATT . In both cases, Table 4 provides the percentage of the number of operations needed by using the proposed algorithm with respect to the number of operations needed by using the respective reference algorithm (first and second to last columns). Overall, the theoretical analysis concludes that in most cases, it is possible to reduce the number of operations. In particular, as could be expected, the ratio is more significant on sequences with a relevant number of TM process activations, since the proposed algorithm realizes a real performance gain only acting on this phase. Nonetheless, the algorithm shows an intrinsic capacity to obtain a performance gain in sequences with a relatively low signal-to-noise ratio, thanks to the mechanism used to determine a sufficient set of relevant points. For instance, in sequence otcbvs 03-l1s3ir-1, the number of activations is comparable among the algorithms, but the gain in terms of reducing the number of operations by using the proposed one is quite significant; in fact, less than 5% of the operations are performed with respect to ATT and PATT, even though the proposed algorithm activates the TM phase a higher number of times than the reference algorithms. Nonetheless, the performance gain becomes less significant when the percentage *S_P_e__* of evaluated points between the reference and proposed algorithms increase. As can be observed, in sequence amcom 17-02-mantruck, where *S_P_e__* is high, the savings in the performed operations is rather low in percentage terms (only 86.32% operations with respect to ATT).

As anticipated, since the theoretical analysis presented in Tables 3 and 4 is based on a series of assumptions (Section 5.1), the computation time per frame has been gathered for each algorithm to be able to compare the real performance (third, fifth and seventh column of the same table). The measured running time inherently includes all of the algorithmic details and, obviously, depends on the implementation of the algorithm. Experiments have been carried out by using a 2.13-GHz Intel Core 2 CPU. Similarly to the theoretical comparison of the number of operations, the third to last and last columns show the ratio between the average time per frame for the proposed algorithm and for the respective reference one (expressed in percentage terms). Furthermore, in this case, percentages smaller than 100% indicate savings in running time. In general, the proposed algorithm shows firm improvements with a growing number of activations. For example, when the reference algorithms do not need any TM activation (e.g., in the OTCBVS dataset in sequences otcbvs 03-l1s2ir-4 and otcbvs 03-l1s3ir-2) or this number is very low (e.g., in the AMCOM dataset in sequences amcom 14-15-mantruck, amcom 16-08-apc, amcom 19-06-apc and amcom 21-17-apc), the speed of the proposed algorithm is comparable to those of the reference ones. On the other hand, it is possible that the low signal-to-noise ratio of the sequences or the presence of similar targets in the scene induces a considerable number of activations; in these cases, e.g., in the AIC dataset in sequences aic ir11-1 and aic ir11-2 or in the OTCBVS dataset in sequence otcbvs 03-l1s2ir-2, the proposed algorithm is able to noticeably boost the performance of the target tracking application.

### 5.3. Tracking Speed vs. Tracking Robustness

After having analyzed the tracking speed of the proposed approach with respect to the reference algorithms, it is worthwhile to extend this analysis to the state-of-the-art algorithms in target tracking scenarios. For this purpose, a set of experimental tests has been carried out by considering several alternative techniques. In particular, they have been selected from a recently implemented benchmark on online tracking [25]. The benchmark is composed of a rather heterogeneous set of target tracking algorithms, but only the most relevant ones for the scope of this work have been chosen for comparison, *i.e.*, the fastest techniques, based on a study presented in [27]. In particular, nine of 29 algorithms have been selected, and all of the them have been tested using their default parameters, like in [25]. The terminology used for identifying the algorithms in this manuscript directly follows the one used in [25]. Moreover, each sequence has been evaluated also in terms of tracking failures in order to find a trade-off between tracking speed and robustness among the various approaches. Furthermore, in this case, various datasets have been considered to test the algorithms in different working conditions.

Results gathered using the considered algorithms are summarized in Table 5. The first two columns identify the name of each sequence and its length. Then, for each considered technique, two columns show the measurements: the first represents the number of tracked frames *T f* for a given sequence (expressed as a percentage value with respect to the length of the sequence), and the latter represents the average tracking speed of the algorithm expressed in frames per second (fps). The first three algorithms are ATT [17], PATT [18] and the proposed one (abbreviated as RATT). Each subsequent technique is identified by the same acronyms used in [25]. Sequences are grouped by dataset, and their average results are highlighted in bold, just as an indication of the performance of the techniques on different datasets. The percentage of tracked frames *T f* for each dataset is computed as the ratio between the sum of the number of correctly tracked frames and the total number of frames in a given dataset (*L*). Similarly, the average speed (*S_A_*) for each dataset is computed as the ratio between the sum of the average frames per second achievable in each sequence of the dataset itself and the total number of frames in the same dataset.

The performance in terms of achievable frames per second for each technique is quite consistent within each dataset. Indeed, the tracking speed for each technique depends both on parameters that are in common within the dataset (such as image resolution) and on parameters that can be set separately for tracking each sequence (e.g., the target window size, which is indicated in Table 2). As expected, the fastest algorithm is KMS [28]. By considering separately the three datasets, the proposed algorithm is second to only KMS, except for the OTCBVS dataset. In fact, in this case, the CSK [29] algorithm provides a higher frame rate than RATT. On the other hand, RATT is faster than KMS on the AMCOM and AIC datasets. The reason resides in the different behavior of the two algorithms. More in detail, CSK is an efficient algorithm that exploits the redundancy that characterize the targets in the process of sampling their features. Generally, OTCBVS sequences are characterized by well-defined target shapes that generate a high contrast with the background. Conversely, the AMCOM and AIC sequences are characterized by a low signal-to-noise ratio; indeed, these sequences present a lot of noise that changes the background around the target frame by frame. This fact indicates that CSK should be preferred in sequences where the target shape and background do not considerably change, such as the ones in the OTCBVS scenario.

The KMS algorithm [28] is the fastest. However, in this case, the speed comes at the cost of a general decrease of robustness. In fact, the KMS algorithm was one of the algorithms with the lowest performance in terms of the number of correctly tracked frames. The only exception is the result in the AIC dataset, where KMS continues to outperform the proposed algorithm in terms of speed, and it is also able to achieve the same result in terms of robustness. In this case, it is worth considering that the test sequences do not present particular challenges in terms of possible failures, due to the static and rather uniform nature of the background; in fact, most algorithms (all, but two) have been able to correctly track all of the frames of the sequences.

Nonetheless, it is worth noticing that in AIC, the relative improvements concerning the speed of the RATT algorithm *versus* the speed of other algorithms are significantly higher than in the other two datasets. In fact, RATT performance in terms of average speed is better, but still comparable to, e.g., the performance of ATT and PATT (in OTCBVS, on average, 492 fps are achieved by RATT, whereas ATT and PATT reach 450 fps and 417 fps, respectively; in AMCOM, on average, 1737 fps are achieved by RATT, whereas ATT and PATT reach 1477 fps and 1725 fps). Instead, in AIC, the improvement in average speed is much better in relative terms than other algorithms; e.g., in RATT, on average, 485 fps can be achieved, whereas only 138 fps and 142 fps can be reached by ATT and PATT, respectively (tripling the improvement in speed with respect to the other datasets). In fact, as indicated in Table 2, the AIC dataset is characterized by a high number of TM activations (*m*), which is the main reason for the computational time savings.

## 6. Conclusions

This paper presented a novel algorithm for improving the speed performance in target tracking applications making use of template matching (TM) techniques in forward-looking infrared images (FLIR). The template matching algorithm is improved by selecting a representative group of points on which it has to be executed, thus reducing both execution time and resources usage. The selection strategy is based on dynamic thresholding and on the results of the target detection (TD) phase. After analyzing the theoretical impact, the paper discusses the results obtained by comparing the proposed technique and the reference implementations on different datasets. Moreover, several alternative techniques are evaluated and included in the performance analysis.

The proposed algorithm showed significant computational performance improvements with respect to reference algorithms, although this came at the cost of introducing some more parameters. Besides target window size and sub-frame size, the two reference algorithms depend only on a probability threshold and on the value of a parameter, whereas the new implementation requires also weights for computing probability and TM values to compare relevant points and a minimum score threshold to get a set of relevant points large enough to be representative of the whole sub-frame without losing essential information.

A different weighting strategy might be devised in the future to improve the precision of the algorithm and to reduce its dependency on arbitrary parameters that may be a hindrance to its use in real-time automatic target tracking applications. For instance, a strategy based on frame features might be used to determine, in a frame-by-frame fashion, the minimum score needed to ensure that the relevant points are a representative set for the current frame. With a similar strategy, a mechanism to automatically set the weights on probability and TM values could be determined in a frame-by-frame fashion. Even though such a strategy might increase the computational complexity of the algorithm, the performance gain found in this paper should be enough to cover the increased complexity.

## Figures and Tables

**Table 1.** List of symbols.

**Symbol**	**Significance**
*n*	current frame number
*m*	number of activations of TM phase in a sequence
*p^n^*^−1^	position of the target at the previous frame
*p̂^n^*	predicted location of the target of interest
*ṗ^n^*	point of coordinates (v,z) belonging to the sub-frame marked as relevant
${ṗ}_{P}^{n}$	point with maximum probability value
${ṗ}_{TM}^{n}$	point with maximum template matching value
${\bar{p}}_{\text{IV F}}^{n}$	point with maximum intensity variation function value
*T^n^*(*p*)	template matching value for a point *p*; see Equation (7)
*P*(*p*)	probability value for a point *p*; see Equation (4)
*ψ*(*p*)	score associated to the point *p*; see Equation (9)
*δ*	adaptive threshold for restricting the computational domain of the TM step
*∊*	minimum score *ψ* (*p*) to be reached by at least one relevant point
*α*	weight for the TM value
*β*	weight for the probability value
Φ	target window area in the computation of *T^n^* (*p*)
*C^n^*(*p*)	correlation output plane value for a point *p*; see Equation (2)
Λ	target window area during the target detection phase (Section 3.1)
*S*_{*P_e_*}_	size of the domain of evaluated points

**Table 2.** Comparison of the number of activations *m* of the TM phase and the number of evaluated points *S*_{*P_e_*}_ among the proposed algorithm and the reference ones, ATT (automatic target tracking) [17] and and PATT (predictive automatic target tracking) [18]. O, OTCBVS dataset; A, AMCOM dataset; AI, AIC dataset; RATT, relevance-based ATT.

**Dataset**			**ATT [17]**		**PATT [18]**		**RATT**		**Δ ATT [17]**	**Δ PATT [18]**
					
**Seq.**	***L***	***S_TW_***	***m***	***S*_{*P_e_*}_**	***m***	***S*_{*P_e_*}_**	***m***	***S*_{*P_e_*}_**	***S*_{*P_e_*}_%**	***S*_{*P_e_*}_%**
**O** 03-l1s1ir-1	154	16 × 30	41	44,649	23	25,047	60	846	1.89%	3.38%
**O** 03-l1s1ir-2	637	7 × 15	4	4,356	4	4,356	F	F	-	-
**O** 03-l1s2ir-1	557	10 × 22	27	29,403	16	17,424	69	648	2.20%	3.72%
**O** 03-l1s2ir-2	339	10 × 20	112	121,968	92	100,188	105	635	0.52%	0.63%
**O** 03-l1s2ir-3	96	9x 18	11	11,979	4	4,356	45	1,548	12.92%	35.54%
**O** 03-l1s2ir-4	24	12 × 30	0	0	0	0	8	18	>100%	>100%
**O** 03-l1s3ir-1	84	11 × 24	31	33,759	29	31,581	37	238	0.70%	0.75%
**O** 03-l1s3ir-2	787	8x 15	33	35,937	0	0	83	373	1.04%	>100%
**O** 03-l1s3ir-3	448	10 × 24	4	4,356	40	43,560	6	26	0.60%	0.06%
**O** 03-l2s4ir-1	270	11 × 30	45	49,005	61	66,429	67	2,080	4.24%	3.13%
**O** 03-l2s6ir-1	323	15 × 28	8	8,712	124	135,036	200	1,382	15.86%	1.02%

**A** 14-15-mantruck	281	10 × 10	6	6,534	3	3,267	19	96	1.47%	2.94%
**A** 16-08-m60	290	13 × 7	60	65,340	18	19,602	32	2,788	4.27%	14.22%
**A** 16-08-apc	80	14 × 8	3	3,267	3	3,267	5	11	0.34%	0.34%
**A** 16-18-apc	300	11 × 8	15	16,335	1	1,089	29	161	0.99%	14.78%
**A** 16-18-m60	103	14 × 8	2	2,178	0	0	1	1	0.05%	>100%
**A** 17-02-mantruck	221	9x 9	2	2,178	16	17,424	39	1,511	69.38%	8.67%
**A** 17-02-bradley	185	9x 9	5	5,445	9	9,801	6	132	2.42%	1.35%
**A** 18-13-m60	227	9x 9	25	27,225	8	8,712	34	550	2.02%	6.31%
**A** 18-16-m60	162	12 × 12	79	86,031	5	5,445	17	150	0.17%	2.75%
**A** 19-06-apc	208	10 × 10	6	6,534	7	7,623	41	367	5.62%	4.81%
**A** 21-17-apc	360	12 × 12	0	0	1	1,089	51	229	>100%	21.03%

**AI** ir11-1	263	21 × 40	93	180,048	110	212,960	147	8,789	4.88%	4.13%
**AI** ir11-2	155	25 × 37	101	195,536	109	211,024	103	2,993	1.53%	1.42%

**Table 3.** A comparison of the number of estimated operations (Θ and Ω) and the real average time per frame *T_A_* among the proposed algorithm and the reference ones ATT [17] and PATT [18]. O, OTCBVS dataset; A, AMCOM dataset; AI, AIC dataset.

**Dataset Seq.**	**ATT [17]**		**PATT [18]**		**RATT**	
		
	**Θ**	***T_A_* (ms)**	**Θ**	***T_A_* (ms)**	**Ω**	***T_A_* (ms)**
**O** 03-l1s1ir-1	44,327,745	1.38	25,529,427	0.6	1,566,894	0.195
**O** 03-l1s1ir-2	7,153,641	0.105	7,153,641	0.075	F	F
**O** 03-l1s2ir-1	18,367,074	0.705	13,108,293	0.615	5,489,448	0.33
**O** 03-l1s2ir-2	51,987,771	1.515	43,297,551	1.41	3,364,434	0.135
**O** 03-l1s2ir-3	4,810,113	0.195	2,347,884	0.165	955,431	0.06
**O** 03-l1s2ir-4	235,224	0.015	235,224	0.09	240,976	0.015
**O** 03-l1s3ir-1	18,614,277	0.69	17,466,471	0.975	842,783	0.225
**O** 03-l1s3ir-2	16,302,330	0.525	7,713,387	0.375	7,733,224	0.42
**O** 03-l1s3ir-3	6,477,372	0.15	25,256,088	0.87	4,393,722	0.135
**O** 03-l2s4ir-1	34,940,565	1.065	46,422,981	1.365	2,690,423	0.3
**O** 03-l2s6ir-1	10,475,091	0.3	116,460,927	4.11	3,333,523	0.24

**A** 14-15-mantruck	4,054,347	0.21	3,404,214	0.165	2,757,862	0.17
**A** 16-08-m60	14,668,830	0.42	6,390,252	0.24	2,848,082	0.20
**A** 16-08-apc	1,512,621	0.18	1,512,621	0.09	785,195	0.09
**A** 16-18-apc	5,798,925	0.255	3,130,875	0.21	2,945,375	0.16
**A** 16-18-m60	1,495,197	0.075	1,009,503	0.075	1,009,726	0.06
**A** 17-02-mantruck	2,516,679	0.15	4,971,285	0.225	2,172,300	0.14
**A** 17-02-bradley	2,689,830	0.21	3,391,146	0.18	1,814,151	0.17
**A** 18-13-m60	6,608,052	0.63	3,627,459	0.15	2,230,301	0.19
**A** 18-16-m60	26,278,659	1.23	3,150,477	0.15	1,592,641	0.14
**A** 19-06-apc	3,338,874	0.165	3,555,585	0.165	2,046,767	0.17
**A** 21-17-apc	3,528,360	0.225	3,840,903	0.165	3,542,997	0.24

**AI** ir11-1	306,883,104	4.88	362,142,352	5.18	4,829,325	0.39
**AI** ir11-2	364,246,784	6.99	392,884,096	5.5	2,891,167	0.24

**Table 4.** A comparison of the number of estimated operations (∆_%_*O*) and real average time per frame ∆_%_*T_A_* with respect to the reference algorithms, ATT [17] and PATT [18]. O, OTCBVS dataset; A, AMCOM dataset; AI, AIC dataset.

**Dataset Seq.**	**RATT/ATT [17]**		**RATT/PATT [18]**	
	
	**Δ_%_*O***	**Δ_%_*T_A_***	**Δ_%_*O***	**Δ_%_*T_A_***
**O** 03-llslir-l	3.53%	14.13%	6.14%	32.50%
**O** 03-llslir-2	-	-	-	-
**O** 03-lls2ir-l	29.89%	46.81%	41.88%	53.66%
**O** 03-lls2ir-2	6.47%	8.91%	7.77%	9.57%
**O** 03-lls2ir-3	19.86%	30.77%	40.69%	36.36%
**O** 03-lls2ir-4	102.45%	100.00%	102.45%	16.67%
**O** 03-lls3ir-l	4.53%	32.61%	4.83%	23.08%
**O** 03-lls3ir-2	47.44%	80.00%	100.26%	112.00%
**O** 03-lls3ir-3	67.83%	90.00%	17.40%	15.52%
**O** 03-l2s4ir-l	7.70%	28.17%	5.80%	21.98%
**O** 03-l2s6ir-l	31.82%	80.00%	2.86%	5.84%

**A** 14-15-mantruck	68.02%	80.02%	8l.0l%	101.84%
**A** l6-08-m60	19.42%	48.03%	44.57%	84.06%
**A** l6-08-apc	51.91%	51.22%	51.91%	102.43%
**A** 16-18-apc	50.79%	62.59%	94.08%	76.00%
**A** l6-l8-m60	67.53%	80.00%	100.02%	80.00%
**A** 17-02-mantruck	86.32%	93.33%	43.70%	62.22%
**A** l7-02-bradley	67.44%	80.02%	53.50%	93.35%
**A** l8-l3-m60	33.75%	30.68%	61.48%	128.88%
**A** l8-l6-m60	6.06%	11.61%	50.55%	95.17%
**A** l9-06-apc	61.30%	101.84%	57.56%	101.84%
**A** 21-17-apc	100.41%	108.39%	92.24%	147.80%

**AI** irll-1	1.57%	7.99%	1.33%	7.53%
**AI** irll-2	0.79%	3.43%	0.74%	4.36%

**Table 5.** A comparison of the percentages of tracked frames and the speed for different tracking algorithms applied to three datasets. TM, template matching.

**Datasets**		**ATT [17]**		**PATT [18]**		**RATT**		**KMS [28]**		**CSK [29]**		**TM**		**PD**		**VR**		**RS**		**CPF [30]**		**MS [31]**		**SMS [20]**	
													
**Sequence**	**L**	***Tf* (%)**	***S_A_* (fps)**	***Tf* (%)**	***S_A_* (fps)**	***Tf* (%)**	***S_A_* (fps)**	***Tf* (%)**	***S_A_* (fps)**	***Tf* (%)**	***S_A_* (fps)**	***Tf* (%)**	***S_A_* (fps)**	***Tf* (%)**	***S_A_* (fps)**	***Tf* (%)**	***S_A_* (fps)**	***Tf* (%)**	***S_A_* (fps)**	***Tf* (%)**	***S_A_* (fps)**	***Tf* (%)**	***S_A_* (fps)**	***Tf* (%)**	***S_A_* (fps)**
otcbvs 03-l1s1ir-1	154	100	322	100	430	100	521	35	3,998	100	207	100	81	100	74	100	65	100	76	100	71	19	60	19	51
otcbvs 03-l1s1ir-2	637	100	546	100	556	64	442	6	6,217	27	1,191	100	98	26	99	25	79	7	69	13	71	2	65	1	35
otcbvs 03-l1s2ir-1	557	100	412	100	427	100	487	33	5,189	41	637	45	98	55	93	100	82	49	61	58	48	28	64	13	26
otcbvs 03-l1s2ir-2	339	100	309	100	319	100	538	3	5,895	34	1,073	100	99	89	86	0	82	2	62	4	91	2	64	2	16
otcbvs 03-l1s2ir-3	96	100	521	100	529	100	560	6	5,764	100	1,044	100	100	100	86	100	77	100	61	76	90	6	64	12	46
otcbvs 03-l1s2ir-4	24	100	575	100	551	100	575	32	3,890	100	209	100	96	36	68	0	65	32	57	100	84	60	61	20	32
otcbvs 03-l1s3ir-1	84	100	414	100	370	100	513	59	5,133	100	537	100	99	100	75	21	73	100	59	100	78	20	63	7	65
otcbvs 03-l1s3ir-2	787	100	444	100	476	100	466	4	6,023	1	1,083	3	91	46	83	46	87	4	62	21	79	3	64	1	39
otcbvs 03-l1s3ir-3	448	100	533	100	385	100	538	4	5,343	96	698	33	80	100	86	100	85	3	68	100	82	73	63	3	134
otcbvs 03-l2s4ir-1	270	100	358	100	324	100	494	26	4,720	100	561	100	91	100	81	100	92	100	75	100	77	25	63	100	82
otcbvs 03-l2s6ir-1	323	100	494	100	171	100	509	91	4,924	100	570	100	90	100	76	17	92	100	60	20	80	80	61	6	27

**OTCBVS TOTAL**	**3719**	**100%**	**450**	**100%**	**417**	**94%**	**492**	**21%**	**5,523**	**51%**	**850**	**63%**	**93**	**68%**	**87**	**57%**	**83**	**35%**	**65**	**46%**	**74**	**25%**	**64**	**12%**	**49**
amcom 14-15-mantruck	281	100	1,626	100	1,754	100	1,745	1	6,663	53	864	75	496	100	474	100	436	100	280	5	124	1	379	1	19
amcom 16-08-m60	290	100	1,220	100	1,550	100	1,648	1	6,250	59	821	100	588	100	422	41	463	100	334	41	115	1	399	14	16
amcom 16-08-apc	80	100	1,709	100	2,000	100	2,011	4	7,557	12	805	100	555	74	416	15	451	62	326	62	110	4	393	9	11
amcom 16-18-apc	300	100	1,515	100	1626	100	1,771	1	6,902	1	830	100	571	27	385	23	430	37	327	10	128	1	384	20	19
amcom 16-18-m60	103	100	2,083	100	2,083	100	2,128	4	7,529	100	816	100	416	46	399	13	426	100	324	78	102	3	364	6	7
amcom 17-02-mantruck	221	100	1,802	100	1,587	100	1,818	1	7,578	41	833	100	463	100	478	100	409	52	335	54	116	2	406	2	13
amcom 17-02-bradley	185	100	1,626	100	1,709	100	1,745	3	7,506	11	770	81	503	43	470	43	426	38	329	59	110	2	390	38	14
amcom 18-13-m60	227	100	966	100	1,802	100	1671	63	7,301	13	791	65	548	9	452	2	451	57	324	2	102	4	402	57	23
amcom 18-16-m60	162	100	612	100	1,802	100	1,826	22	7,114	31	805	77	497	100	468	74	415	56	321	56	103	9	389	93	9
amcom 19-06-apc	208	100	1,754	100	1,754	100	1,745	10	7,609	29	604	100	535	38	473	100	436	59	326	96	110	2	391	3	15
amcom 21-17-apc	360	100	1,587	100	1,754	100	1,541	1	6,768	1	763	1	536	100	420	100	410	1	322	1	112	1	392	1	15

**AMCOM TOTAL**	**2,417**	**100%**	**1,477**	**100%**	**1,725**	**100%**	**1,737**	**9%**	**7,045**	**29%**	**792**	**76%**	**527**	**70%**	**441**	**62%**	**431**	**56%**	**322**	**34%**	**114**	**2%**	**391**	**20%**	**16**
aic ir11-1	263	100	151	100	145	100	472	100	3,496	100	162	100	73	100	72	100	72	100	60	100	81	38	65	23	2
aic ir11-2	155	100	115	100	138	100	508	100	3,769	100	196	100	72	100	85	100	68	100	50	100	67	12	62	19	2

**AIC TOTAL**	**418**	**100%**	**138**	**100%**	**142**	**100%**	**485**	**100%**	**3,597**	**100%**	**175**	**100%**	**73**	**100%**	**77**	**100%**	**71**	**100%**	**56**	**100%**	**76**	**28%**	**64**	**22%**	**2**
